# Effect of Dietary Seaweed Supplementation in Cows on Milk Macrominerals, Trace Elements and Heavy Metal Concentrations

**DOI:** 10.3390/foods10071526

**Published:** 2021-07-02

**Authors:** Eric E. Newton, Ásta H. Pétursdóttir, Gunnar Ríkharðsson, Corentin Beaumal, Natasa Desnica, Konstantina Giannakopoulou, Darren Juniper, Partha Ray, Sokratis Stergiadis

**Affiliations:** 1 Department of Animal Sciences, School of Agriculture Policy and Development, University of Reading, Earley Gate, P.O. Box 237, Reading RG6 6EU, UK; eric.newton@pgr.reading.ac.uk (E.E.N.); k_giannakopoulou@yahoo.com (K.G.); d.t.juniper@reading.ac.uk (D.J.); p.p.ray@reading.ac.uk (P.R.); 2 Matís Ltd., Vínlandsleid 12, 113 Reykjavik, Iceland; corentin.beaumal@etu.unistra.fr (C.B.); natasa@matis.is (N.D.); 3 BSSL, Agricultural Society of South Iceland, Austurvegur 1, 800 Selfoss, Iceland; gunnar@bssl.is; 4 ECPM—Ecole Europeenne de Chimie, Polymeres et Materiaux, Université de Strasbourg, 25 Rue Becquerel, 67087 Strasbourg, France

**Keywords:** milk, iodine, minerals, seaweed, *Laminaria digitata*, *Ascophyllum nodosum*, Icelandic cow

## Abstract

This study investigated the effect of seaweed supplementation in dairy cow diets on milk yield, basic composition, and mineral concentrations. Thirty-seven Icelandic cows were split into three diet treatments: control (CON, no seaweed), low seaweed (LSW, 0.75% concentrate dry matter (DM), 13–40 g/cow/day), and high seaweed (HSW, 1.5% concentrate DM, 26–158 g/cow/day). Cows were fed the same basal diet of grass silage and concentrate for a week, and then were introduced to the assigned experimental diets for 6 weeks. The seaweed mix of 91% *Ascophyllum nodosum*: 9% *Laminaria digitata* (DM basis), feed, and milk samples were collected weekly. Data were analyzed using a linear mixed effects model, with diet, week, and their interaction as fixed factors, cow ID as random factor, and the pre-treatment week data as a covariate. When compared with CON milk, LSW and HSW milk had, respectively, less Se (−1.4 and −3.1 μg/kg milk) and more I (+744 and +1649 μg/kg milk), while HSW milk also had less Cu (−11.6 μg/kg milk) and more As (+0.17 μg/kg milk) than CON milk. The minimal changes or concentrations in milk for Se, Cu, and As cannot be associated with any effects on consumer nutrition, but care should be taken when I-rich seaweed is fed to cows to avoid excessive animal I supply and milk I concentrations.

## 1. Introduction

Seaweed is an underexploited potential animal feed source that has recently gained increased attention due to its high concentration of specific minerals, macronutrients, and bioactive compounds, spearheaded by indications that certain seaweed species have been shown to markedly reduce enteric methane emissions [1,2,3,4,5]. Seaweed farming and wild harvesting have a number of benefits, including faster growth rates from traditional crops farmed on land and less of a vulnerability to the meteorological effects of climate change, therefore reducing the increasing competition between food and feed production from traditional land-based agricultural production [6]. Global seaweed production has increased by almost 27% between 2011 and 2015, resulting in a total output of 30 million tons at the end of this period, and continues to grow, with the vast majority of seaweed being from farmed aquaculture [7]. Given the current state of harvesting seaweed, and its potential benefits on animal nutrition and health, there is an increasing interest towards alternative applications for the growing industry, such as the sustainable seaweed supplementation of animal diets which may yield potential benefits for ruminant health and nutrition along with resulting benefits to human health [8,9,10].

Of the several seaweed species that have been previously explored, *Ascophyllum nodosum* and *Laminaria digitata* have been identified as potential candidates for experimental animal feeding [11,12,13]. *A. nodosum* is a brown cold-water alga which is found in much of the Northern Atlantic Ocean, including Norway, the United Kingdom, Iceland, and the eastern seaboard of the United States and Canada [14,15]. *A. nodosum* is either gathered by hand (e.g., Scotland and Ireland) or by mechanical harvesting (e.g., Norway and Iceland) and is one of the main species harvested in Europe; its use as a bio-stimulant for agricultural opportunities have been recently researched and it is currently used in much of phycological industrial applications, such as fertilizer and alginate production, along with a function as an animal feed supplement [16,17,18]. *L. digitata*, is a less harvested but still common seaweed found within the Northern Atlantic Ocean, with an estimated total harvested amount annually (<150 tons in the wild) being lower than *A. nodosum* in Ireland [19]. *L. digitata* is one of the most exploited types of seaweed off the coast of France, where it has been harvested for alginates at around 50,000 tons a year as of 2011 [20]. These two species (i) have been shown to illicit a positive effect on rumen function, animal health, energy utilization, and milk quality and safety [3,10,13,21,22,23,24,25]; (ii) are good sources of minerals, such as iodine (I), calcium (Ca), phosphorus (P), selenium (Se), magnesium (Mg), and zinc (Zn) representing excellent candidates for feed mineral supplementation [26,27,28]; (iii) are excellent sources of essential amino acids for the animal, such as theanine, valine, methionine, isoleucine, leucine, phenylalanine, lysin, histidine, and arginine—of which, for many, dairy serves as a source of [27,29,30]; and (iv) are readily available in Europe, Scandinavia, and the eastern seaboard of North America [14,15].

While previous findings regarding the effect of seaweed supplementation in ruminant diets on animal health and rumen function are promising, the impact on milk quality should also be considered. Milk and dairy products are rich in minerals and are large suppliers of I, Ca, P, Se, Mg and Zn in human diets [31,32]. These minerals can exert positive effects on human health as they are associated with reduced risk of cardiometabolic diseases and other non-communicable diseases, therefore providing a source of nutrition and the potential to reduce healthcare expenses [33]. A common characteristic of most seaweeds is the high mineral content, and therefore, supplementation of dairy cow diets with this aquacultural product may influence mineral concentrations in the milk [28]. Previous work has found that dietary supplementation of cow diets with *A. nodosum* has increased milk I concentrations by approximately 309%, to 481 μg/L average across three periods, when offered at 113 g per cow per day, and by approximately 671% to 1370 μg/L when cows were offered 170 g per head per day [10,34]. In another study, supplementation of dairy cow diets with a blend of seaweeds, including *Ulva rigida*, *Laminaria ochroleuca*, *Saccharina latissima*, *Saccorhiza polyschides*, *Mastocarpus stellatus*, and *Sargassum muticum* resulted in higher milk I content, indicating that dietary supplementation of seaweed could be used as a potential strategy to increase milk I content [10,24]. However, seaweed may contain heavy metals, including cadmium (Cd), lead (Pb), mercury (Hg), copper (Cu), molybdenum (Mo), and arsenic (As) [35,36,37]. Some of these heavy metals are considered contaminants in the food chain and there is a requirement to maintain their concentrations in foods below certain thresholds, although there are currently no published maximum statutory limits for As, Cd, or Hg in milk in Europe; while Pb is limited to 20 μg/kg milk [24,38,39]. Brown macroalgae in particular may contain high concentrations of total As but usually with low levels of the toxic inorganic As (e.g., *A. nodosum*), however, *L. digitata* is a notable exception to this as it can contain high concentrations of both [40,41]. Supplementation of dairy cow diets with a mixture of *U. rigida* (green seaweed), *S. muticum* (brown seaweed) and *S. polyschides* (brown seaweed), increased As content in milk while Cd and Pb concentration was unaffected [24]. 

While there is increasing interest of several seaweed species as animal feed, high seasonal and between-species variation in mineral and chemical composition of seaweed species [42,43,44] suggests a need for vigilant screening of seaweeds, as well as the development of corresponding animal feeding strategies. This will ensure that seaweed supplementation to dairy cow diets improves or at least maintains milk yield, quality, and safety characteristics. Therefore, the present study aimed to (i) investigate the effect of feeding a mixture of seaweed (9% *Laminaria digitata* + 91% *Ascophyllum nodosum*) to dairy cows at different dietary inclusion rates (0.75% and 1.5% of the concentrate dry matter (DM)) on milk yield and basic composition, along with the concentrations of macrominerals, trace elements and heavy metals, and (ii) estimate the impact that the consumption of milk from seaweed-fed cows may have on consumer mineral intakes.

## 2. Materials and Methods

### 2.1. Experimental Design

The current study was conducted during the winter indoor period at Stóra-Ármót farm, Selfoss, Iceland. Animal procedures were reviewed by The Icelandic Food and Veterinary Authority and confirmed that the experiment did not require a license according to the regulation no. 460/2017. Thirty-seven lactating dairy cows of the Icelandic breed were blocked into three groups of 11 to 13 cows each, balanced for parity, lactation stage, milk yield and milk contents of fat, protein, and somatic cell count (SCC). Before the experiment began, all cows received a basal diet made up of 4.8–11.4 kg DM concentrate feed (ingredients list presented in Supplementary Materials, Table S1) according to milk yield, topped up with ad libitum supply of grass silage. Each group was assigned to one of three experimental diets (i) without seaweed supplementation (control, CON), (ii) with seaweed supplementation at 0.75% seaweed in concentrate, DM basis (low seaweed, LSW; 13–40 g seaweed/cow/day), and (iii) 1.5% seaweed in concentrate, DM basis (high seaweed, HSW; 26–158 g seaweed/cow/day). The seaweed mix comprised of 91% *Ascophyllum nodosum* and 9% *Laminaria digitata*, on DM basis. These seaweeds were selected because they represent species with high commercial potential as they are abundant and easy to access; the dietary inclusion rate was based on not exceeding maximum levels of heavy metals according to the European Commission. Commission Regulation for maximum levels for As in animal feed, where 2 mg/kg diet DM of inorganic As in the seaweed mixture was the limiting factor [45]. The chemical composition of silage and concentrate are shown in Table 1 and mineral composition of silage, concentrate, and concentrate with seaweed are shown in Table 2. The average chemical composition and mineral composition of the three experimental diets are presented in Tables S2 and S3, respectively, in the Supplementary Materials. Animal data (estimated bodyweight, lactation stage, parity) are presented in Table 3. Feed intake was calculated as described by Butler et al. [46], using estimated bodyweight and milk yield.

The experiment was carried out over a 7-week period between December 2018 and January 2019. All animals were fed the CON diet for two weeks before the commencement of the 7-week period. The starting week was used as a covariate, where all cows were fed the basal diet, and this was followed by 6-week measurement period where animals were offered experimental diets. Seaweed was gradually introduced to diets. In week 1 of the measurement period, seaweed was provided at approximately 0.25% (13 g/cow/day) and 0.50% (26 g/cow/day) of concentrate DM for LSW and HSW groups, respectively. In weeks 2 to 5, seaweed was provided at 0.75% (19–40 g/cow/day) and 1.5% (79–158 g/cow/day) of concentrate DM for LSW and HSW groups, respectively. In week 6, dietary inclusion rate of seaweed returned to approximately 0.25% (13 g/cow/day) and 0.50% (26 g/cow/day) of concentrate DM for LSW and HSW groups, respectively. Cows were milked twice daily. Milk samples were collected from each cow at the end of each experimental week during the morning and evening milkings, and composite milk samples were stored frozen (at −18 °C) in a 50 mL polypropylene tube. Samples of grass silage were collected once a week (*n* = 7) during the experimental period and immediately frozen at −18 °C. Samples of concentrate without seaweed were collected in experimental weeks 1, 3, and 5 (*n* = 3), while samples of concentrate with seaweed were collected in weeks 3 and 5 (*n* = 2). All feed samples were stored at −18 °C until further analysis.

### 2.2. Analysis of Milk and Feed for Chemical Composition

The basic composition (fat, protein, casein, lactose, urea, free fatty acids (FFA)) and somatic cell count (SCC) of milk was analyzed using Fourier Transform Infrared Spectroscopy (Combifoss 6000, FOSS, Hilleroed, Denmark) in the laboratories of Auðhumla (Selfoss, Iceland). Samples of silages and concentrates were analyzed for chemical composition (crude protein, CP; fat; sugar; starch; sugar, neutral detergent fiber, NDF; acid detergent fiber, ADF; water soluble carbohydrates, WSC; single cell protein, SCP; indigestible NDF, iNDF; neutral detergent cellulase digestible organic matter, NCDG) at the laboratories of Efnagreining (Hvanneyri, Iceland).

### 2.3. Quantification of Mineral Concentrations in Milk and Feed

Concentrations of macrominerals, trace elements (except for I) and heavy metals in milk, silage and concentrate feed were quantified according to NMKL method 186 [48], using an Ultra wave Acid Digestion System (Milestone Inc., Sorisole, Italy) for the digestion of samples. An Agilent 7900 quadrupole inductively coupled plasma mass spectrometer (ICP-MS) (Agilent Technologies, Singapore) was used. It was combined with an ultra-high matrix introduction (UHMI) system with a quartz cyclonic spray chamber and MicroMist nebulizer (Glass Expansion, Weilburg, Germany). Concentrations of I in milk and feed samples were quantified according to previously published methods by [49] and British Standards Institution Publication (BS EN 17050:2017), respectively, using ICP-MS (Agilent 7000, Agilent, Singapore). For Sn, Cd, Cr, Ni, Pb and Hg, the majority of the individual measurements (88% for Sn, 96% for Cd, 59% for Cr, 53% for Ni, 82% for Pb and 92% for Hg) were below the limits of quantification (LOQ; Sn, 0.266 μg/kg milk; Cd, 0.099 μg/kg milk; Cr, 0.696 μg/kg milk; Ni, 1.457 μg/kg milk; Pb, 0.335 μg/kg milk; Hg, 0.243 μg/kg milk); and the results of these elements were thus not included in statistical analysis. The scatter plots of all measurements of mineral concentrations in the three experimental treatments, and in relation to LOQ, are presented in supplementary Figure S1 (macrominerals), Figure S2 (trace elements) and Figure S3 (heavy metals). Transfer efficiencies from feed to milk were calculated as follows: 100 × (milk mineral concentration (ug/kg milk) × milk output (kg/d)/diet mineral concentration (ug/kg dry matter) × feed intake (kg dry matter/day)).

### 2.4. Statistical Analysis

Data were analyzed using a mixed effects model in Minitab 18. In the model, diet, experimental week, and their interaction were used as fixed factors, while cow was set as the random factor [50]. Measurements from the week before the 6-week measurement period, when all cows were fed the same basal diet, were used as a covariate in the model. Normality of residuals were evaluated visually and, while most data showed no deviation from normality, SCC, milk I content, and I intake were log10 transformed prior to analysis so that their residuals were normalized. Fischer’s least significance difference test (*p* < 0.05) was used for pairwise comparison of the means, where the mixed effect model showed a significant effect of diet, experimental week, or their interaction.

## 3. Results

### 3.1. Animal and Diet Parameters

The experimental groups were balanced for parity, lactation stage and bodyweight (Table 3). Parity ranged 1–4, 1–5, and 1–5, in CON, LSW and HSW groups, respectively. Lactation stage in weeks ranged 1–42, 1–68, and 1–47 in CON, LSW and HSW groups, respectively. The dietary treatment influenced seaweed intake which increased from CON to LSW, and LSW to HSW cows, in line with the experimental design (Table 3). Seaweed intake significantly differed between experimental groups, averaging 0 g, 12.8 g, and 50.2 g for CON, LSW, and HSW groups, respectively (Table 3). The DMI, forage:concentrate ratio, silage intake and concentrate intake varied by experimental week (Table 3; Table S4).

### 3.2. Milk Yield, Basic Composition, and Efficiency

Milk from HSW group cows had 4.1% and 2.2% less protein (g/100 g) and 4.3% and 2.6% less casein (g/100 g), when compared with CON and LSW milk, respectively (Table 3). There was a significant effect of dietary treatment on milk protein and casein concentration. However, milk production, and other compositional and efficiency parameters were not influenced by dietary supplementation of seaweed (Table 3). Milk yield, milk composition (e.g., contents of protein, casein, lactose, whey protein, urea, and FFS), and feed efficiency varied with experimental week (Table 3; Table S4). Individual significant differences between weeks are presented in detail in the Supplementary Materials (Table S4). There was no significant diet × sampling week interaction on milk production, milk basic composition, or efficiency parameters (Table 3).

### 3.3. Milk Mineral Concentrations

Dietary supplementation of seaweed influenced milk concentrations of Cu, I, Se, and As, with CON milk having a 32.5% higher Cu concentration compared to HSW milk (Table 4). When compared with CON milk, I concentrations were greater in LSW (+90.5%) and HSW milk (+200.8%); while HSW milk had higher (+57.8%) concentrations of I than LSW milk (Table 4). However, the trend was the opposite for Se concentration in milk. When compared with CON milk, concentrations of Se were lower in LSW milk (−6.0%) and HSW milk (−13.4%); while HSW contained less Se (−8.5%) than LSW milk (Table 4). The concentration of As in HSW milk was higher compared with LSW and CON milk (+28.8% and +36.7%, respectively) (Table 4).

**Figure 1:**
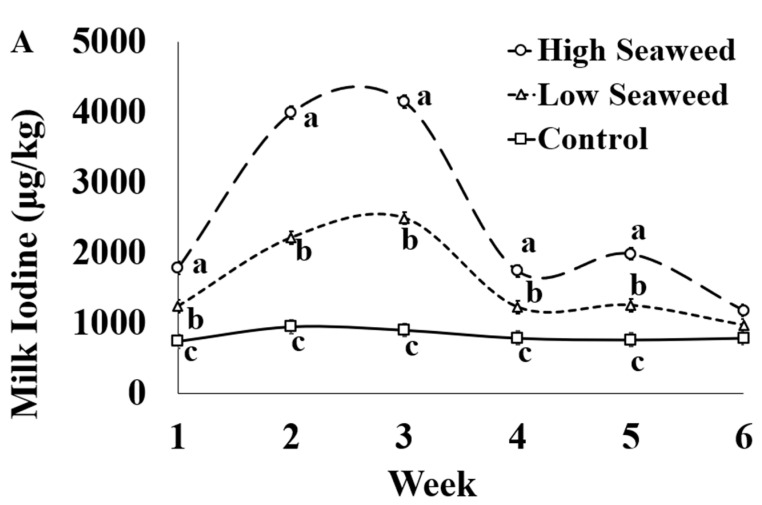

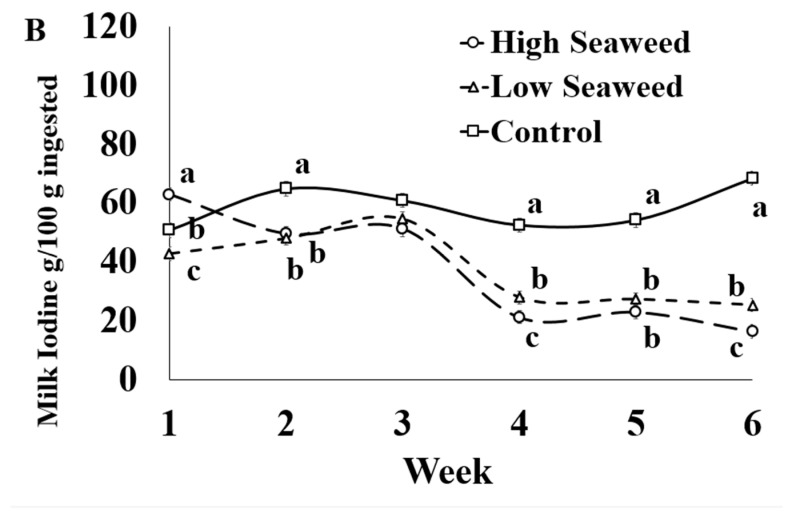
Interaction means ± SE (error bars) for the effects of dietary treatment (Control, no seaweed, CON; Low seaweed, 0.75% concentrate DM, LSW; High Seaweed, 1.5% concentrate DM, HSW) and week on the concentration of iodine in milk (μg/kg; panel (**A**); *p* <0.001) and transfer efficiency (g milk/100 g ingested; panel (**B**); *p* < 0.001). Means for diet treatments within a week with different letters are significantly different according to Fisher’s Least Significant Difference test (*p* < 0.05).

The effect of sampling week was significant for all macrominerals, trace elements, and heavy metals assessed (Table S5). Individual significant differences between weeks are presented in detail in the Supplementary Materials (Table S5). The I concentration in milk was influenced by the dietary treatment × sampling week interaction (Figure 1A). Milk I concentration was highest in HSW milk, intermediate in LSW and lowest in CON milk throughout seaweed supplementation period. Their relative difference in milk I concentrations between all experimental groups was higher during weeks 2 and 3 compared with the rest of weeks. HSW contained significantly more I across the experiment than LSW, except for Week 6 where there was not difference between the experimental groups.

### 3.4. Estimated Mineral Transfer Efficiencies from Feed to Milk

There was a significant effect of dietary treatment on the estimated transfer efficiency of Cu, I, Se, and Co. The transfer efficiency of Cu was higher (+0.5 μg/kg intake) in CON milk than HSW milk (Table 5). Transfer efficiency of I was higher (+21 μg/kg intake) in CON milk when compared to LSW and HSW milk (Table 5). Similarly, transfer efficiency of Se was higher (+0.7 μg/kg intake and +1.0 μg/kg intake) in CON milk than in LSW and HSW milk, respectively (Table 5). The transfer efficiency of Co was higher (+0.005 μg/kg intake and +0.009 μg/kg intake) in CON milk than LSW and HSW milk, respectively (Table 5). 

The effect of sampling week was significant on the transfer efficiency of all assessed macrominerals, trace elements, and heavy metals and individual significant differences between weeks are presented in detail in the Supplementary Materials (Table S6).

The only significant effects of the dietary treatment × sampling week interaction mineral transfer efficiency from feed to milk was for I (Table 5). In Week 1, I transfer efficiencies were highest in HSW milk, intermediate in CON milk and lowest in LSW milk. Between Weeks 2 and 6, I transfer efficiencies were higher in CON milk than in LSW and HSW milk (except for Week 3), while LSW also resulted on higher I transfer efficiencies than HSW milk in Weeks 4 and 6 (Figure 1B).

## 4. Discussion

### 4.1. Effect of Seaweed Supplementation on Milk Yield, Basic Composition and Efficiency Parameters

In the present study, seaweed supplementation of dairy cow diets did not affect productivity, efficiency, and the basic composition of milk, thus agreeing with previous studies feeding *Ascophyllum nodosum* and *Undaria pinnatifida* [10,51,52]. Given that main drivers for productivity, production efficiency and milk composition are animal breed, intakes, and types of forages and concentrates [53,54], it is likely that the relatively small amount of seaweed supplementation to dairy cow diets (0 to 158 g/cow/day) in the present study was not adequate to cause any impact on these parameters. In contrast, Singh et al. [55] reported that *S. wightii* supplementation at 20% to concentrate DM showed increased milk production in dairy cows. This discrepancy could be attributed to the much larger degree of supplementation as they administered approximately 955 g of seaweed per cow per day and indicated that there might be unidentified bioactive substances within the seaweed that may have positively affected milk yield at such high supplementation rates. The only milk composition parameters affected by seaweed supplementation in the present study were milk protein and casein contents, which were both reduced in case of HSW diets. This is different than the studies of Hong, Kim, Jin, Lee, Choi and Lee [51] and Chaves Lopez, Serio, Rossi, Mazzarrino, Marchetti, Castellani, Grotta, Fiorentino, Paparella and Martino [52], which saw no change in milk protein concentrations with increasing brown seaweed by-products or *A. nodosum* supplementation, respectively. However, the differences in the present study were numerically marginal as HSW contained only 1.3 g/kg less protein and 1.0 g/kg less casein than CON milk.

### 4.2. Effect of Seaweed on Milk Mineral Concentrations and Estimated Mineral Transfer Efficiencies from Feed to Milk

#### 4.2.1. Trace Elements 

The reduced Cu concentration in milk with increased seaweed supplementation in the present study is in contrast with other studies that showed seaweed supplementation did not impact milk Cu content [24]. In the current study, reduced Cu concentration was found in HSW milk despite the minimal difference in dietary intakes of Cu between experimental groups (614.5 mg/cow/day for CON, 413.1–934.1 mg/cow/day; 602.3 mg/cow/day for LS, 376.1–932.9 mg/cow/day; and 615.1 mg/cow/day for HSW, 403.6–926.9 mg/cow/day). This might indicate that the appearance of Cu into milk might be mediated by physiological or metabolic processes rather than simply Cu intake. Milk Cu concentrations are unaffected by high Cu intakes, but when Cu intakes are restricted below requirement there is a commensurate decrease in milk Cu concentrations [56]. Although Cu availability has not been assessed in this study, a possible explanation might be that Cu availability from the CON was higher than that in seaweed-supplemented diets (in line with the lower Cu transfer efficiency observed in the current study). However, it should be noted that Cu regulation is more complicated than a simple input/output relationship and involves several organ systems [56]. In general, differences between studies may also originate from the use of different species of seaweed, known to affect mineral concentrations [28], which was a mix of *A. nodosum* and *L. digitata* in this study and a mix of *Ulva rigida*, *Sargassum muticum*, and *Saccorhiza polyschides* in the study of Rey-Crespo, López-Alonso and Miranda [24], as well as the dietary supplementation level (158 g/cow/day maximum in the present study for the HSW group vs. 100 g/cow/day in study by Rey-Crespo, López-Alonso and Miranda [24]). 

In the current study, seaweed supplementation in dairy cow diets increased I concentrations in milk, which is in line with the findings from previous work that investigated the effect of feeding *A. nodosum* [10,52,57] and kelp powder or *Thallus laminariae* to dairy cows on milk I concentration [58]. Concentrations of I in raw milk are primarily influenced by diet I concentrations, but in-feed goitrogenic compounds, husbandry practices, and mammary gland hygiene management (teat-dipping) are also determinant factors [10,59,60]. Seaweed is a known rich source of I [26,28] and in the present study, I intake across the experimental period was 35.0 mg/cow/day (21.2–48.5 mg/cow/day), 107 mg/cow/day (60.4–163.6 mg/cow/day), and 178.7 mg/cow/day (60.4–281.1 mg/cow/d) for CON, LSW, and HSW cows, respectively. Therefore, LSW and HSW cows ingested 3.1 and 5.1 times more I, respectively, than CON cows, which could explain the higher concentration of I in the milk from LSW and HSW cows. 

In the present study, the diet I concentration was 2.4 mg/kg DM for CON, 7.5 mg/kg DM for LSW and 12.3 mg/kg DM for HSW cows. Given the maximum permitted dietary I concentration is 5 mg/kg DM [61], the I concentration in CON, LS and HS diets was 48%, 150%, and 246% of maximum permitted I concentration, respectively. Notably, at the peak of seaweed supplementation (weeks 2, 3, 5), dietary supply of I to LSW and HSW cows temporarily exceeded 2.2 and 4.0 times of the maximum permitted supply. This indicates that care should be taken when seaweed is supplemented in dairy cow diets for long periods because small amounts of I-rich seaweed may supply far higher amounts of I in dairy cow diets than the maximum permitted intakes. The upper tolerable limit dietary I for cattle is reported to be 50 mg/kg of diet DM [62]. At an average DMI of 14.4 kg/day, as calculated in the present study, the maximum tolerable limit for I intake would be 720 mg/cow/day. Therefore, although LSW and HSW diets exceeded permitted dietary supplementation of I for cattle, I intake by LSW and HSW cows in the current study was, respectively, on average 15% and 25% of the upper tolerable limit for cattle, and never exceeded the 40% of upper tolerable limit. Although the dietary I supply in the current study was much lower than the upper tolerable limit, after one week adaptation in seaweed diets and two weeks after peak seaweed supplementation, the I transfer from feed to milk dropped from 55% and 51% to 28% and 21% in LSW and HSW cows, respectively. In mammals, excessive I intake triggers the Wolff-Chaikoff effect reducing I absorption from the gut to blood [63]. A similar mechanism may not be excluded in dairy cows and therefore, the rapid increase in I supply may have triggered a reduced absorption of I and subsequent supply in the mammary gland and/or a down regulation of the Na+/I- symporter system in the mammary gland; both of which would reflect in reduced I concentrations in milk despite the high intakes. After the end of the experiment, I was monitored for 3 more weeks in the cows that consumed LSW and HSW diets and the transfer efficiencies of I returned to the pre-supplementation levels (52% and 57% for LSW and HSW cows, respectively), only a week after removal of seaweed from the diet, which may indicate that this impact is reversible, at least after the exposure duration to LSW and HSW diets investigated in the present study, when I supply returns to recommended levels; possibly because the Na+/I- symporter system returns to pre-high dose levels.

The reduced Se concentration in milk with increased seaweed supplementation in the present study is in contrast with the findings of a previous study that reported that seaweed supplementation did not impact milk Se concentration [24]. Even though Se intake was not different between experimental groups in the current study (8.4 mg/cow/day for CON (5.7–11.5 mg/cow/day), 8.3 mg/cow/day for LSW (5.3–11.5 mg/cow/day), and 8.5 mg/cow/day for HSW (5.7–11.4 mg/cow/day)), there was still a decrease in milk Se concentration in the LSW and HSW groups. This indicates that the resulting concentrations might be influenced by physiological or metabolic processes rather than being a direct effect of Se intake. Milk Se concentrations are influenced by cow supplementation and feed types (varying widely between different areas [64,65], and has been shown to be increased (albeit short-lived) with dietary Se increases [56]. Another explanation could be in that an increase in sulfur supplied from seaweed, as sulphate is a typical component of marine algal polysaccharides, may antagonize selenium absorption, or that the form of Se found within the treatment feed may affect uptake [24,66,67]. Reduced transfer efficiency might be a consequence of an interaction between Se and Se antagonists thus reducing the uptake and transfer of selenium from feed into milk. The differences between Rey-Crespo, López-Alonso and Miranda [24] and this study can also be explained via the differing species and amount fed to the cows, as described above for Cu. 

The decreased Co transfer efficiency with increased seaweed supplementation in the present study was not reflected in the Co content between the experimental groups. This is likely due to differences between the groups and the total Co transfer efficiency results numerically extremely small—as the difference between the highest and lowest transfer efficiency is 0.009%.

#### 4.2.2. Heavy Metals 

Increasing seaweed supplementation in cow diets increased As concentrations, thus being in line with Rey-Crespo, López-Alonso and Miranda [24]. This is expected, as the most prominent heavy metal in algae is As, hence the EU there is relevant regulation regarding the maximum amount in algae in feed [41]. As intake across the experimental period was 6.0 mg/cow/day for CON (2.8–14.81 mg/cow/day), 6.7 mg/cow/day for LSW (3.1–15.2 mg/cow/day), and 9.3 mg/cow/day for HSW (4.5–15.6 mg/cow/day). The higher dietary intake of As when seaweed was fed is the most possible reason for the increased As content in milk, as As intake leads to increased milk As content [68]. Any amount of inorganic As (which is more toxic than organic As [69]) in feed or product is recommended to be avoided, and US NRC reports that the maximum tolerable dosage for cattle is 50 mg/kg diet DM [70]. At an average DMI of 14.4 kg/day in the present study, the maximum tolerable limit for inorganic As intake would be 720 mg/cow/day, which is 46 times higher than the maximum As intake in the present study (15.6 mg/cow/day). In the present study, the analysis did not differentiate between organic or inorganic As and diets were designed to supply less than the maximum limits of As in dairy cow diets (2 mg/kg inorganic As and 40 mg/kg total As in the seaweed mixture [45]). 

### 4.3. Nutritional Implications of Milk from Seaweed-Fed Cows for Consumers (I, Cu, Se, As)

Milk is a good source of several macrominerals and trace elements and this has particular importance for different demographics which may have higher requirements or rely more on milk for the supply of minerals across infancy, adolescence, and adulthood [71]. In the present study, the concentrations of Cu, I, Se, and As were affected by seaweed supplementation in dairy cow diets and this would have an effect on consumer intakes of these minerals when consuming milk from seaweed-fed cows. To assess the impact of seaweed supplementation of dairy diets on consumers’ mineral intakes, the mineral intakes from the milk of experimental groups was calculated by multiplying the recorded average milk intakes in Iceland (kg of liquid milk per person per day) with the concentrations of I, Cu, Se, and As (ug, or mg, per kg milk). Following that, the calculated mineral intakes were compared against the nutritional recommendations (reference nutrient intakes (RNI) and upper limits (UL)) by the Icelandic Directorate of Health [72] to assess the % contribution that milk would provide to the RNI, but also investigate whether consumption of any minerals exceeds UL, when milk from different experimental groups would be consumed.

The average consumption of milk in Iceland is 285 g/day, according the most recent available milk sale records (2020) from Icelandic Dairies Association (Samtök afurðastöðva í mjólkuriðnaði); based on this, CON, LSW, and HSW milks would cover 2.7–4.5%, 2.3–3.9%, and 2.0–3.4% of the RNI for Cu in children <10 years of age, respectively; 1.5–1.9%, 1.3–1.7%, and 1.1–1.5% in adolescents and adults ≥10 years of age, respectively; and 1.0–1.4%, 0.9–1.2%, and 0.8–1.0% in nursing mothers and pregnant women, respectively. Given that this amount does not represent a considerable proportion of RNI for Cu for all age groups at Icelandic levels of consumption, it is unlikely that these differences will have a relevance to consumers’ nutrition and health.

Based on the above-referenced average consumption of milk in Iceland, the CON, LSW and HSW milks would cover 196–470%, 373–895% and 589–1413% of the RNI for I in children <10 years of age, respectively; 157%, 299% and 471% of the RNI for I in adolescents and adults ≥10 years of age, respectively; and 117–134%, 224–256% and 353–404% in nursing mothers and pregnant women, respectively. Even consumption of CON milk from the present study, and under the stated milk intakes in Iceland, would provide more than the required I to the population to meet their RNI for I. This is of particular importance because I deficiency prevails globally, occurring in 435.5 million (56.9% of the population) and almost 2 billion (35.2% of the population) people in Europe and globally, respectively [73]. Although in Iceland this was not a public health issue for years, more recent studies have highlighted that specific demographics (including pregnant women) had suboptimal I intake and have associated this with the reduction in milk, dairy and fish consumption [74]. The results for the CON milk in the present study reinforce the important role that milk can play in providing the required amounts of I in human diets. Interestingly, the milk I content of the CON milk (822 μg/kg) was substantially higher than that in countries neighboring Iceland (e.g., 331 μg/kg milk in conventional UK milk [75] 232 μg/L in winter low-fat Norwegian milk [76]; and 670 μg/kg in Irish milk that involved pre- and post-milking teat dipping in I-containing solution [77]). These higher concentrations may be due to the experimental farm being on the banks of the Ölfusá River, a body of water that carries glacial water (commonly rich in I) and at close proximity to the sea (~20 km) [78,79]. Coastal areas have more I in the soil and subsequently produce forage that may also have higher I concentrations [80]. The potential effect of Icelandic cow genetics may not be excluded as it is known that breed can also be a driver for milk I concentrations [75,81]. The combination of even standard milk being rich in I, and the relatively high average consumption of milk in Iceland (26th in the world and 23rd in Europe, [82]), contributed to a high calculated contribution of milk towards the RNI for I.

However, supplementation of dairy diets with seaweed would exacerbate an excessive I intake. The upper limit for I in adults is 600 μg/day, and high consumption of I may induce hypothyroidism, in which susceptible individuals fail to adapt to the acute Wolff-Chaikoff effect, or hyperthyroidism in which vulnerable individuals increase thyroid hormone production due to the rich I substrate, inducing thyrotoxicosis [83]. When comparing these intakes with the recommended UL for adults [72], consumption of CON, LSW and HSW milk would provide 39%, 75% and 118% of the upper limit. This highlights that, although high in I, CON and LSW milk would not provide an amount that would be considered a risk (at a consumption rate of 285 g/day) but drinking milk from the HSW group at the average Icelandic intake levels would exceed the UL for I. From a different perspective, the UL for I intake in adults would be reached by drinking 730 g of CON milk, 383 g of LSW milk, or 243 g of HSW milk. Although Icelandic guidelines were not available for UL in children and adolescents, EFSA [84] recommends that UL for children <10 years of age to be 200–300 μg/day and UL for adolescents (10–17 years of age) to be 450–500 μg/day. Based on this, the UL can be reached by children drinking 243–365 g of the CON milk, 128–192 g of the LSW milk and 81–122 g of the HSW milk. For adolescents, the UL can be reached by children drinking 548–608 g of the CON milk, 287–319 g of the LSW milk and 182–203 g of the HSW milk. It is important however to note that in the present study, I concentrations in LSW and HSW averaged 7.5 and 12.3 mg/kg DM, respectively, while CON diet contained 2.4 mg/kg DM. Such high diet I concentrations as in LSW and HSW groups are unlikely to be provided in commercial herds because I supplementation in dairy diets ought to be less than 5 mg/kg DM [85]. Although these diets do not represent potential commercial examples, and therefore it is unlikely that milk with such high I content would reach the Icelandic market, the findings highlight that extreme care should be taken when seaweed is supplemented to dairy cow diets because even small amounts of I-rich seaweed can not only exceed I allowances in dairy cow diets, but also drastically increase milk I concentrations and potentially pose a nutritional risk to the consumers.

Based on the above-referenced average consumption of milk in Iceland, CON, LSW, and HSW milks would cover 22–44%, 21–42%, and 19–38% of the RNI for Se in children <10 years of age, respectively; 12–17%, 11–16%, and 11–14% in adolescents and adults ≥10 years of age, respectively; and 11%, 11%, and 10% for nursing mothers and pregnant women, respectively. Although milk appears to be among the main suppliers of Se in the Icelandic diets, and seaweed supplementation in dairy diets influences milk Se concentrations, the numerical differences are rather small. As a result, the consumption of CON, LSW or HSW milk would marginally differentiate the proportionate contribution of milk to RNI for Se and it is unlikely that consuming milk from different groups would impact consumer nutrition and health.

As is a toxic heavy metal and should generally be avoided in foodstuffs, as previous nutritional research council reports have not found a biochemical process in which As is required, and that the concept of As essentiality is still to be researched [86]. The WHO provisional guideline recommendation is that As intake should not exceed 10 μg/L in drinking water [87]. The milks in the present study contained 0.46 μg/kg (CON), 0.48 μg/kg (LSW) and 0.62 μg/kg (HSW), thus all having extremely low As concentrations, being only 4.6%, 4.8% and 6.2% of the maximum recommended concentrations in water. Notably, this recommendation for milk As content is paired with the Tropical Agriculture Association’s (TAA) published requirements for humans living in temperate conditions to drink 3 L of water per day [88]; which would provide a recommended maximum As supply of 30 μg/day. In the present study, considering above-referenced average consumption of milk in Iceland, CON, LSW, and HSW milks would account for 0.13, 0.14, and 0.18 μg/day, respectively, which represents 0.4–0.6% of the maximum recommended As intake. Therefore, milk cannot be considered a source of As and the consumption of milk of any experimental group is not associated with any potential As-related risks in human nutrition and health; a finding which also aligns with previous studies using other seaweeds (*Ulva rigida*, *Sargassum muticum*, *Saccorhiza polyschides*, fed at 80.0:17.5:2.5 ratio at 100 g per animal per day) [24]. In addition, it should be noted that the present study has not differentiated between organic and inorganic As, a parameter that also influences toxicity with inorganic As posing a higher toxicity [69]. Therefore, the intakes of inorganic As could be smaller given that a fraction of As in milk, might be present as organic As, however, since the total As concentration is so low a distinction between inorganic and organic As is not relevant from a toxicological point of view [89]. In general, milk is not a source of heavy metals in human diets as only traces were detected, mostly below an already extremely low LOQ, which are far below the maximum recommended levels for milk, and this is not expected to be associated with effects on human health. 

## 5. Conclusions

Seaweed supplementation (9% *Laminaria digitata* + 91% *Ascophyllum nodosum*) did not affect cow productivity or milk basic composition, except for a small reduction in milk protein and casein content. However, seaweed supplementation reduced contents of Cu and Se in milk and increased contents of I and As in milk. The increases in milk I and As contents are likely due to the higher dietary supply of I and As, although the lower concentrations of Cu and Se seem to be more associated with a reduction in their transfer efficiencies from diet to milk, when seaweed was included in cows’ diets. Despite the lower milk Cu and Se contents when seaweed was supplemented in dairy diets, the subsequent calculated contribution of the different milks on Cu and Se reference nutrient intakes (based in Icelandic population milk intakes and nutritional guidelines) were marginally different and unlikely to be related with any effect on consumers nutrition or health. This study further emphasizes the main role that milk plays in I supply as even consumption of the control milk would provide more than the required I to the population to meet their RNI for I. However, the findings also showed that extreme care should be taken when seaweed is supplemented to dairy diets because even small amounts of I-rich seaweed can exceed the cow dietary I allowances but also drastically increase milk I concentrations and potentially pose a nutritional risk for consumers. Seaweed supplementation of dairy diets increased As concentrations in milk but milk from all experimental groups contained only traces of As and consumption cannot not be associated with any potential As-related risks in human nutrition and health.

## Figures and Tables

**Table 1:** Means, standard deviation (SD), minimum and maximum values for the chemical composition of silage and concentrate used in the animal trial.

	Silage				Concentrate ^1^			
Chemical Composition (g/kg Dry Matter)	Mean	SD	Min	Max	Mean	SD	Min	Max
Dry Matter (g/kg fresh)	301	7.0	290	309	895	1.2	894	897
Ash	70	2.4	67	74	89	2.7	83	93
NCDG ^2^	768	12.2	750	780	NM ^3^	NM ^3^	NM ^3^	NM ^3^
Crude Protein	166	11.6	143	178	213	3.3	203	220
Neutral Detergent Fiber	501	15.5	483	525	118	6.6	103	129
Acid Detergent Fiber	301	13.6	285	328	564	46.0	501	647
Single Cell Protein	111	5.8	101	118	NM ^3^	NM ^3^	NM ^3^	NM ^3^
Indigestible Neutral Detergent Fiber	87	12.1	78	113	NM ^3^	NM ^3^	NM ^3^	NM ^3^
Sugar	47	9.9	30	62	NM ^3^	NM ^3^	NM ^3^	NM ^3^
Fat	59	4.7	54	65	26	1.2	24	28
Ammonia	0.7	0.14	0.5	0.9	NM ^3^	NM ^3^	NM ^3^	NM ^3^
Starch	NM ^3^	NM ^3^	NM ^3^	NM ^3^	276	9.4	252	286
Water-soluble Carbohydrates	NM ^3^	NM ^3^	NM ^3^	NM ^3^	136	12.2	115	160

^1^ Compound feed in the form of pellet comprising of soybean, wheat, corn, barley, sugar beet flour, molasses, shell lime, hard fat, mono-calcium phosphate, magnesium phosphate, salt, and mineral/vitamin supplement in ratios as presented in Table S1 in the Supplementary Materials. ^2^ Neutral Detergent Cellulase Digestible Organic Matter. ^3^ not measured.

**Table 2:** Means, standard deviation (SD), minimum and maximum values for the mineral composition of silage, concentrate and seaweed used in the animal trial.

	Silage				Concentrate ^1^				Concentrate with Seaweed ^2^			
Minerals (mg/kg Dry Matter)	Mean	SD	Min	Max	Mean	SD	Min	Max	Mean	SD	Min	Max
Aluminum (Al)	552	698.8	164	2107	510	166.1	408	701.6	419	50.3	383	454
Arsenic (As)	0.07	0.045	0.04	0.17	0.77	0.526	0.41	1.373	1.06	0.106	0.98	1.13
Cadmium (Cd)	0.02	0.005	0.01	0.02	0.08	0.022	0.05	0.097	0.11	0.004	0.11	0.12
Calcium (Ca)	4373	484.8	3859	5220	14,945	342.8	14,632	15,311	14,979	446.1	14,663	15,294
Chromium (Cr)	37	40.7	12	128	26.1	8.0	19.2	34.78	22	3.0	20	24
Cobalt (Co)	0.66	0.461	0.37	1.68	3.60	0.785	2.82	4.393	2.67	0.184	2.54	2.80
Copper (Cu)	11	1.4	9	13	71	10.2	60	80.88	57	3.8	55	60
Iodine (I)	0.22	0.138	0.14	0.53	4.2	0.33	3.6	4.499	18	6.2	8	26
Iron (Fe)	1468	1317.2	685	4392	917	260.9	723	1212.0	760	50.1	725	795
Lead (Pb)	0.09	0.081	0.04	0.27	0.37	0.084	0.29	0.460	0.20	0.000	0.20	0.20
Magnesium (Mg)	1939	182.1	1757	2260	4936	250.8	4783	5225	4670	166.4	4552	4788
Manganese (Mn)	73	11.6	54	89	181	36.8	145	218.0	193	28.1	173	213
Mercury (Hg)	0.01	0.000	0.01	0.01	0.00	0.00	0.00	0.00	0.003	0.0035	0.000	0.005
Molybdenum (Mo)	0.67	0.366	0.37	1.46	2.2	0.39	1.8	2.517	1.94	0.209	1.80	2.09
Nickel (Ni)	13	9.6	6	34	11.9	2.31	9.3	13.72	11.3	1.06	10.5	12.0
Phosphorus (P)	3091	632.2	2635	4429	7208	257.1	5912	7372	7005	416.3	6710	7299
Potassium (K)	20,399	2102.5	17,581	24,024	11,991	739.6	11,254	12,733	12,814	935.2	12,152	13,475
Selenium (Se)	0.17	0.055	0.10	0.28	0.94	0.052	0.89	0.993	0.98	0.039	0.96	0.01
Sodium (Na)	1067	220.6	730	1331	3279	157.6	3111	3424	3082	184.4	2951	3212
Tin (Sn)	0.09	0.038	0.06	0.17	0.12	0.017	0.10	0.130	0.13	0.004	0.13	0.14
Zinc (Zn)	43	5.1	36	51	153	29.9	126	185.2	130	13.7	120	139

^1^ Compound feed in the form of pellet comprising of soybean, wheat, corn, barley, sugar beet flour, molasses, shell lime, hard fat, mono-calcium phosphate, magnesium phosphate, salt, and mineral/vitamin supplement (their proportional contribution is presented in Table S1 in the Supplementary Materials. ^2^ Containing 15 g/kg DM seaweed mixture on DM basis. Seaweed mixture was made of 91% *Ascophyllum nodosum* + 9% *Laminaria digitata*.

**Table 3:** Means, standard error (SE) and ANOVA *p*-values for the effect of the dietary treatment (Control, no seaweed, CON; Low Seaweed, 0.75% concentrate DM, LS; High Seaweed, 1.5% concentrate DM, HS) on animal data, milk production and basic composition and efficiency parameters.

	Diet				ANOVA *p*-Values ^1^		
Parameters	CON *n* = 66	LSW *n* = 78	HSW *n* = 78	SE	Diet	Week	Diet × Week
**Animal Data**							
Parity	2.0	2.1	2.3	0.15			
Lactation weeks	20.0	24.0	21.9	1.79			
Bodyweight (kg)	445	446	450	5.0			
**Animal Diet**							
Dry Matter Intake ^2^ (kg/d)	14.3	14.3	14.5	0.08	0.075	0.041	0.943
Forage:concentrate	44.7	45.4	44.8	1.80	0.942	<0.001	0.793
Silage Intake (kg DM/d)	6.35	5.43	6.47	0.254	0.946	<0.001	0.776
Concentrate Intake (kg DM/d)	8.01	7.85	8.02	0.251	0.872	<0.001	0.844
Seaweed Intake (g DM/d)	0.00 ^c^	12.8 ^b^	50.2 ^a^	0.004	<0.001	<0.001	<0.001
**Milk Production**							
Yield (kg/d)	25.3	24.9	26.5	0.60	0.097	0.041	0.943
ECMY ^3^ (kg/d)	27.0	25.9	27.1	0.70	0.399	0.133	0.775
**Milk Composition**							
Fat (g/100 g)	4.56	4.46	4.35	0.082	0.157	0.843	0.878
Protein (g/100 g)	3.33 ^a^	3.27 ^a^	3.20 ^b^	0.027	0.004	<0.001	0.632
Casein (g/100 g)	2.43 ^a^	2.39 ^a^	2.33 ^b^	0.234	0.006	<0.001	0.694
Lactose (g/100 g)	4.58	4.62	4.63	0.030	0.517	<0.001	0.767
Whey Protein (g/100 g)	0.90	0.88	0.87	0.008	0.111	<0.001	0.627
Urea (mmol/L)	6.37	6.13	3.08	0.139	0.283	<0.001	0.109
Free Fatty Acids (mmol/L)	0.80	0.87	0.91	0.038	0.144	<0.001	0.929
Fat:Protein	1.37	1.37	1.36	0.028	0.931	0.198	0.579
Somatic Cell Count (×10^3^/mL)	181	206	193	65.1	0.965	0.255	0.699
**Efficiency (g/kg DMI)**							
Feed Efficiency	1753	1730	1807	29.6	0.134	0.022	0.899
Fat Efficiency	80.4	76.8	77.8	2.15	0.461	0.335	0.637
Protein Efficiency	58.3	56.2	57.3	0.86	0.191	0.244	0.894

^1^ Significances were declared at *p* < 0.05. Means for diet treatment within a row with different letters are significantly different according to Fisher’s Least Significant Difference test (*p* < 0.05). ^2^ calculated as described by Butler et al. [46]: DMI (kg/day) = 0.025 LW (kg) + 0.125 milk yield (kg/day). ^3^ Energy Correct Milk Yield = milk yield (kg) × (0.01 + 0.0122 milk fat (g/kg) + 0.0077 milk protein (g/kg) + 0.053 milk lactose (g/kg)) [47].

**Table 4:** Means, standard error (SE) and ANOVA *p*-values for the effect of the dietary treatment (Control, no seaweed, CON; Low seaweed, 0.75% concentrate DM, LSW; High Seaweed, 1.5% concentrate DM, HSW) on milk mineral concentrations.

	Diet				ANOVA *p*-Values ^1^		
Minerals	CON *n* = 66	LSW *n* = 78	HSW *n* = 78	SE	Diet	Week	Diet × Week
**Macrominerals (mg/kg)**							
Calcium (Ca)	1129	1076	1053	29.7	0.192	<0.001	0.797
Magnesium (Mg)	110.4	103.0	99.2	4.30	0.179	0.021	0.481
Phosphorus (P)	881.8	866.8	851.0	26.72	0.708	<0.001	0.892
Potassium (K)	1471	1433	1423	40.2	0.661	<0.001	0.711
Sodium (Na)	432.9	435.2	403.0	20.31	0.422	0.033	0.525
**Essential Trace Elements (μg/kg)**							
Copper (Cu)	47.3 ^a^	40.9 ^ab^	35.7 ^b^	3.05	0.034	<0.001	0.364
Iron (Fe)	223.9	224.1	223.9	9.72	1.000	0.020	0.337
Iodine (I)	821.5 ^c^	1565.3 ^b^	2470.8 ^a^	60.98	<0.001	<0.001	<0.001
Manganese (Mn)	27.5	28.4	27.4	1.06	0.717	0.009	0.173
Molybdenum (Mo)	52.5	51.9	49.4	1.62	0.346	<0.001	0.296
Nickel (Ni)	2.49	1.60	1.40	0.440	0.182	<0.001	0.105
Selenium (Se)	23.2 ^a^	21.8 ^b^	20.1 ^c^	0.50	<0.001	<0.001	0.987
Zinc (Zn)	4720	4683	4406	125.5	0.137	<0.001	0.842
**Non-Essential Trace Elements (μg/kg)**							
Aluminum (Al)	63.7	57.3	60.1	4.53	0.577	<0.001	0.202
Cobalt (Co)	0.52	0.48	0.43	0.029	0.088	<0.001	0.140
**Heavy Metals (μg/kg)**							
Arsenic (As)	0.455 ^b^	0.483 ^b^	0.622 ^a^	0.0416	0.013	<0.001	0.102

^1^ Significances were declared at *p* < 0.05. Means for diet treatment within a row with different letters are significantly different according to Fisher’s Least Significant Difference test (*p* < 0.05).

**Table 5:** Means, standard error (SE) and ANOVA *p*-values for the effect of the dietary treatment (Control, no seaweed, CON; Low seaweed, 0.75% concentrate DM, LSW; High Seaweed, 1.5% concentrate DM, HSW) on estimated transfer efficiency of minerals from feed into milk.

	Diet				ANOVA *p*-Values ^1^		
Minerals (g in Milk/100 g Ingested)	CON *n* = 66	LSW *n* = 78	HSW *n* = 78	SE	Diet	Week	Diet × Week
**Macrominerals**							
Calcium (Ca)	19.7	18.4	18.0	0.67	0.170	<0.001	0.830
Magnesium (Mg)	8.7	7.9	7.6	0.51	0.294	<0.001	0.846
Phosphorus (P)	29.2	28.1	27.8	0.99	0.590	<0.001	0.944
Potassium (K)	16.2	16.1	15.8	0.72	0.912	<0.001	0.834
Sodium (Na)	33.3	32.4	29.9	1.74	0.341	0.031	0.488
**Essential Trace Elements**							
Copper (Cu)	0.20 ^a^	0.17 ^a,b^	0.15 ^b^	0.145	0.042	<0.001	0.308
Iron (Fe)	0.04	0.04	0.04	0.002	0.638	<0.001	0.364
Iodine (I)	58.7 ^a^	37.7 ^b^	37.5 ^b^	1.70	<0.001	<0.001	<0.001
Manganese (Mn)	0.04	0.04	0.04	0.002	0.950	<0.001	0.279
Molybdenum (Mo)	6.8	6.6	6.3	0.23	0.378	<0.001	0.436
Nickel (Ni)	0.05	0.03	0.02	0.011	0.215	<0.001	0.087
Selenium (Se)	7.2 ^a^	6.5 ^b^	6.2 ^b^	0.22	0.007	<0.001	0.961
Zinc (Zn)	8.5	8.4	7.9	0.23	0.128	<0.001	0.690
**Non-Essential Trace Elements**							
Aluminum (Al)	0.03	0.02	0.03	0.002	0.563	<0.001	0.252
Cobalt (Co)	0.044 ^a^	0.039 ^a,b^	0.035 ^b^	0.0025	0.037	<0.001	0.128
**Heavy metals**							
Arsenic (As)	0.22	0.20	0.19	0.018	0.679	<0.001	0.252

^1^ Significances were declared at *p* < 0.05. Means for diet treatment within a row with different letters are significantly different according to Fisher’s Least Significant Difference test (*p* < 0.05).

## Data Availability

The raw data presented in this study are available on request from the corresponding author.

## Supplementary Materials

The following are available online at https://www.mdpi.com/article/10.3390/foods10071526/s1, Table S1: Ingredient composition (g/100 g dry matter) of the concentrate feed fed during the animal trial, Table S2: Means, standard deviation (SD), minimum and maximum values for the average chemical composition of the three experimental diets used in the animal trial, Table S3: Means, standard deviation (SD), minimum and maximum values for the average mineral concentrations of the three experimental diets used in the animal trial, Table S4: Means, standard error (SE) and ANOVA p-values for the effect of the dietary treatment (Control, no seaweed, CON; Low Seaweed, 0.75% concentrate DM, LSW; High Seaweed, 1.5% concentrate DM, HSW) on animal data, milk production and basic composition and efficiency parameters. Table S5: Means, standard error (SE) and ANOVA p-values for the effect of the dietary treatment (Control, no seaweed, CON; Low seaweed, 0.75% concentrate DM, LSW; High Seaweed, 1.5% concentrate DM, HSW) on mineral composition within the resulting milk, Table S6: Means, standard error (SE) and ANOVA p-values for the effect of the dietary treatment (Control, no seaweed, CON; Low seaweed, 0.75% concentrate DM, LSW; High Seaweed, 1.5% concentrate DM, HSW) on transfer efficiency of minerals from feed to milk per week, Figure S1: Scatter plots of all measurements of macromineral concentrations in milk samples collected throughout the study from the three experimental groups (▢, control, no seaweed; ×, Low seaweed, 0.75% concentrate DM; ◯, High Seaweed, 1.5% concentrate DM), Figure S2: Scatter plots of all measurements of trace element concentrations in milk samples collected throughout the study from the three experimental groups (▢, control, no seaweed; ×, Low seaweed, 0.75% concentrate DM; ◯, High Seaweed, 1.5% concentrate DM). The horizontal dotted lines represent limits of quantification for each element, Figure S3: Scatter plots of all measurements of heavy metal concentrations in milk samples collected throughout the study from the three experimental groups (▢, control, no seaweed; ×, Low seaweed, 0.75% concentrate DM; ◯, High Seaweed, 1.5% concentrate DM). The horizontal dotted lines represent limits of quantification for each element.
