# Supplementary material for: Effect of Dietary Seaweed Supplementation in Cows on Milk Macrominerals, Trace Elements and Heavy Metal Concentrations
Source: Foods. 2021 Jul 2;10(7):1526. doi: 10.3390/foods10071526 (PMC8307245; doi:10.3390/foods10071526)
Supplement: Supplementary file 1 [file foods-10-01526-s001.zip › foods-1255344-supplementary.pdf]

foods MDPI

Supplementary Material

Table S1

Ingredient composition (g/100g dry matter) of the concentrate feed fed during the animal trial.

Soybean	31.0
Wheat	21.0
Corn	15.1
Barley	10.45
Sugar Beet Flour	8.0
Molasses	6.0
Shell Lime	2.5
Hard Fat	2.0
Mono-calcium Phosphate	1.5
Magnesium Phosphate	1.5
Salt	0.7
Minerals/Vitamins	0.25

foods MDPI

Table S2

Means, standard deviation (SD), minimum and maximum values for the average chemical composition (g/kg dry matter (DM)) of the three experimental diets (Control, no seaweed, CON; Low seaweed, 0.75% concentrate DM, LSW; High Seaweed, 1.5% concentrate DM, HSW) used in the animal trial

	Control				Low-Seaweed				High-Seaweed			
**Chemical composition (g/kg DM)**	**Mean**	**SD**	**Min**	**Max**	**Mean**	**SD**	**Min**	**Max**	**Mean**	**SD**	**Min**	**Max**
Dry Matter (g/kg fresh)	62.9	0.4	62.4	63.4	61.8	0.4	61.3	62.3	63.4	0.4	62.9	63.9
Ash	8.0	0.3	7.6	8.4	7.9	0.3	7.5	8.3	8.1	0.3	7.6	8.5
Crude Protein	19.2	0.7	17.6	20.1	18.9	0.7	17.3	19.8	19.3	0.7	17.7	20.2
Neutral Detergent Fiber	28.9	1.1	27.3	30.6	28.7	1.0	27.0	30.3	28.8	1.1	27.2	30.5
Acid Detergent Fiber	44.6	3.1	40.4	50.4	43.9	3.1	39.8	49.6	44.9	3.2	40.7	50.8
Fat	4.1	0.3	3.7	4.5	4.0	0.3	3.7	4.4	4.1	0.3	3.7	4.4

Table S3

Means, standard deviation (SD), minimum and maximum values for the average mineral concentrations (mg/kg dry matter (DM)) of the three experimental diets (Control, no seaweed, CON; Low seaweed, 0.75% concentrate DM, LSW; High Seaweed, 1.5% concentrate DM, HSW) used in the animal trial

	Control				Low-Seaweed				High-Seaweed			
**Minerals (mg/kg DM)**	**Mean**	**SD**	**Min**	**Max**	**Mean**	**SD**	**Min**	**Max**	**Mean**	**SD**	**Min**	**Max**
Aluminum (Al)	517	320.1	258	1365	521	324.2	245	1501	527	317.0	252	1477
Arsenic (As)	0.41	0.202	0.22	0.92	0.47	0.200	0.23	0.97	0.64	0.169	0.32	1.00
Cadmium (Cd)	0.05	0.010	0.03	0.07	0.05	0.011	0.03	0.07	0.05	0.010	0.03	0.07
Calcium (Ca)	10206	820.5	8714	11755	10182	900.5	7787	11951	10371	896.1	8066	12565
Chromium (Cr)	31	19.4	16	82	31	19.6	15	91	30	19.2	15	89
Cobalt (Co)	2.20	0.446	1.54	3.08	2.18	0.467	1.30	3.12	2.21	0.457	1.34	3.13
Copper (Cu)	42	6.4	31	58	42	6.9	28	58	42	6.8	28	59
Iodine (I)	2.4	0.37	1.6	3.0	7.5	1.57	4.5	10.9	12.3	3.91	4.4	20.2
Iron (Fe)	1147	618.7	709	2829	1153	625.8	707	3113	1152	613.1	711	3054
Lead (Pb)	0.23	0.059	0.15	0.33	0.23	0.061	0.13	0.33	0.23	0.059	0.13	0.34
Magnesium (Mg)	3152	1080.8	606	4147	3155	1067.3	570	4292	3199	1077.6	572	4367
Manganese (Mn)	128	18.8	96	172	128	19.6	89	173	128	19.4	90	173
Mercury (Hg)	0.005	0.0008	0.003	0.006	0.005	0.0008	0.003	0.006	0.005	0.0008	0.002	0.006
Molybdenum (Mo)	1.44	0.274	1.07	1.93	1.43	0.283	0.94	1.97	1.44	0.277	0.97	1.99
Nickel (Ni)	12	4.8	8	25	12	4.9	8	26	12	4.8	8	26
Phosphorus (P)	5402	444.9	4572	6337	5373	455.6	4302	6510	5397	452.2	4265	6623
Potassium (K)	15842	1106.2	13803	18517	16011	1187.8	13793	19788	16292	1200.0	13836	19897
Selenium (Se)	0.58	0.071	0.44	0.71	0.58	0.077	0.39	0.73	0.58	0.076	0.40	0.74
Sodium (Na)	2272	192.2	1833	2645	2308	213.1	1653	2797	2463	234.9	1814	3038
Tin (Sn)	0.10	0.021	0.08	0.15	0.10	0.021	0.07	0.15	0.10	0.021	0.07	0.15
Zinc (Zn)	100	15.7	75	140	99	16.6	69	141	100	16.5	70	141

foods MDPI

Table S4

Means, standard error (SE) and ANOVA P-values for the effect of the dietary treatment (Control, no seaweed, CON; Low Seaweed, 0.75% concentrate DM, LSW; High Seaweed, 1.5% concentrate DM, HSW) on animal data, milk production and basic composition and efficiency parameters.

	Week							ANOVA P-values
**Parameters**	**1 n=37**	**2 n=37**	**3 n=37**	**4 n=37**	**5 n=37**	**6 n=37**	**SE**	**Week**
**Animal diet**								
Estimated DMI [2] (kg/d)	14.4^{ab}	14.5^{a}	14.4^{b}	14.3^{b}	14.4^{b}	14.3^{b}	0.05	0.041
Forage:concentrate	43.0^{e}	43.7^{de}	44.6^{cd}	45.2^{bc}	46.0^{b}	47.2^{a}	1.03	<0.001
Silage Intake (kg DM/d)	6.17^{e}	6.27^{de}	6.35^{cd}	6.44^{bc}	6.57^{b}	6.72^{a}	0.146	<0.001
Concentrate Intake (kg DM/d)	8.25^{a}	8.18^{a}	8.02^{b}	7.90^{bc}	7.79^{c}	7.62^{d}	0.155	<0.001
Seaweed Intake (g DM/d)	13.2^{c}	51.5^{a}	50.9^{a}	49.8^{a}	49.0^{a}	37.3^{b}	2.37	<0.001
**Milk production**								
Yield (kg/d)	25.9^{ab}	26.2^{a}	25.5^{b}	25.2^{b}	25.4^{b}	25.2^{b}	0.40	0.041
ECMY [3] (kg/d)	26.8^{ab}	27.2^{a}	26.7^{ab}	26.3^{b}	26.6^{ab}	26.4^{b}	0.45	0.133
**Milk composition**								
Fat (g/100g)	4.44	4.46	4.50	4.48	4.46	4.42	0.062	0.843
Protein (g/100g)	3.23^{c}	3.32^{c}	3.28^{ab}	3.26^{bc}	3.28^{ab}	3.31^{a}	0.019	<0.001
Casein (g/100g)	2.34^{c}	2.37^{bc}	2.39^{b}	2.35^{c}	2.38^{b}	2.45^{a}	0.017	<0.001
Lactose (g/100g)	4.58^{cd}	4.64^{ab}	4.61^{bc}	4.60^{bcd}	4.57^{d}	4.66^{a}	0.020	<0.001
Whey Protein (g/100g)	0.89^{b}	0.86^{c}	0.89^{b}	0.91^{a}	0.90^{b}	0.86^{c}	0.005	<0.001
Urea (mmol/L)	5.90^{d}	5.52^{e}	6.58^{b}	6.80^{a}	6.13^{c}	6.22^{c}	0.093	<0.001
FFA [4] (mmol/L)	0.81^{c}	0.61^{d}	0.90^{b}	0.88^{bc}	0.93^{b}	1.04^{a}	0.031	<0.001
Fat:Protein	1.37^{a}	1.38^{a}	1.37^{ab}	1.38^{a}	1.36^{ab}	1.33^{b}	0.020	0.198
SCC [5] (x10^3/ml)	144^{b}	221^{ab}	179^{ab}	272^{a}	168^{ab}	176^{ab}	49.8	0.255
**Efficiency (g/kg DMI)**								
Milk Efficiency	1779^{ab}	1798^{a}	1758^{bc}	1743^{c}	1756^{bc}	1746^{bc}	19.8	0.022
Fat Efficiency	78.6^{ab}	79.7^{a}	78.8^{ab}	77.8^{ab}	78.1^{ab}	76.9^{b}	1.42	0.335
Protein Efficiency	57.1^{ab}	57.8^{a}	57.4^{ab}	56.5^{b}	57.4^{ab}	57.5^{ab}	0.58	0.244

[1] Significances were declared at $P < 0.05$. Means for diet treatment within a row with different letters are significantly different according to Fisher's Least Significant Difference test ($P < 0.05$)

[2] Dry Matter Intake

[3] Energy Correct Milk Yield = milk yield (kg) × [0.01 + 0.0122 milk fat (g/kg) + 0.0077 milk protein (g/kg) + 0.053 milk lactose (g/kg)] [88]

[4] Free Fatty Acids

[5] Somatic Cell Count

Table S5

Means, standard error (SE) and ANOVA P-values for the effect of the dietary treatment (Control, no seaweed, CON; Low seaweed, 0.75% concentrate DM, LSW; High Seaweed, 1.5% concentrate DM, HSW) on milk mineral concentrations

	Week						ANOVA P-values	
	1	2	3	4	5	6		
**Minerals**	**n=37**	**n=37**	**n=37**	**n=37**	**n=37**	**n=37**	**SE**	**Week**
**Macrominerals (mg/kg)**								
Calcium (Ca)	1127^{ab}	968^{d}	1150^{a}	1149^{a}	1073^{bc}	1049^{c}	26.5	<0.001
Magnesium (Mg)	116.9^{a}	92.2^{c}	107.7^{ab}	107.7^{ab}	101.2^{bc}	99.4^{bc}	5.26	0.021
Phosphorus (P)	864.2^{bc}	812.6^{c}	903.3^{ab}	922.2^{a}	835.2^{c}	861.4^{bc}	22.45	<0.001
Potassium (K)	1469^{ab}	1337^{d}	1512^{a}	1531^{a}	1418^{bc}	1387^{cd}	33.49	<0.001
Sodium (Na)	473.1^{a}	378.1^{c}	432.8^{ab}	411.3^{bc}	423.5^{abc}	423.6^{abc}	20.98	0.033
**Essential trace elements (µg/kg)**								
Copper (Cu)	47.7^{a}	43.1^{b}	48.7^{a}	40.0^{b}	34.6^{c}	33.8^{c}	2.16	<0.001
Iron (Fe)	237.4^{a}	231.6^{a}	237.4^{a}	217.6^{ab}	202.1^{b}	217.9^{ab}	9.37	0.02
Iodine (I)	1254.9^{c}	2383.6^{b}	2514.1^{a}	1251.3^{c}	1333.2^{c}	978.2^{d}	52.51	<0.001
Manganese (Mn)	26.1^{b}	27.3^{ab}	29.2^{a}	28.6^{a}	26.0^{b}	29.4^{a}	0.97	0.009
Molybdenum (Mo)	48.0^{c}	48.0^{c}	53.2^{b}	63.1^{a}	46.1^{c}	49.1^{c}	1.42	<0.001
Nickel (Ni)	1.06^{b}	1.12^{b}	1.32^{b}	4.60^{a}	2.05^{b}	0.81^{b}	0.546	<0.001
Selenium (Se)	23.0^{a}	22.7^{a}	23.6^{a}	22.1^{a}	19.3^{b}	19.6^{b}	0.58	<0.001
Zinc (Zn)	4794^{b}	4609^{bc}	5233^{a}	4482^{cd}	4323^{de}	4175^{e}	109.5	<0.001
**Non-essential trace elements (µg/kg)**								
Aluminum (Al)	59.0^{b}	43.5^{b}	85.1^{a}	56.7^{b}	58.6^{b}	59.4^{b}	6.11	<0.001
Cobalt (Co)	0.56^{a}	0.43^{bc}	0.57^{a}	0.48^{b}	0.44^{bc}	0.39^{c}	0.024	<0.001
**Heavy metals (µg/kg)**								
Arsenic (As)	0.63^{a}	0.53^{abc}	0.59^{ab}	0.52^{bc}	0.38^{d}	0.47^{cd}	0.041	<0.001

[1] Significances were declared at $P < 0.05$. Means for diet treatment within a row with different letters are significantly different according to Fisher's Least Significant Difference test ($P < 0.05$)

Table S6

Means, standard error (SE) and ANOVA P-values for the effect of the dietary treatment (Control, no seaweed, CON; Low seaweed, 0.75% concentrate DM, LSW; High Seaweed, 1.5% concentrate DM, HSW) on transfer efficiency of minerals from feed to milk per week

	Week						ANOVA P-values	
**Minerals**	**1**	**2**	**3**	**4**	**5**	**6**		
**(g in milk/100g ingested)**	**n=37**	**n=37**	**n=37**	**n=37**	**n=37**	**n=37**	**SE**	**Week**
**Macrominerals (mg/kg)**								
Calcium (Ca)	19.2^{bc}	16.8^{e}	20.1^{ab}	20.4^{a}	18.2^{cd}	17.6^{de}	0.51	<0.001
Magnesium (Mg)	6.13^{b}	4.57^{c}	5.02^{bc}	23.18^{a}	4.86^{bc}	4.72^{bc}	0.546	<0.001
Phosphorus (P)	30.3^{b}	27.8^{c}	28.9^{bc}	30.7^{a}	24.3^{d}	28.1^{c}	0.79	<0.001
Potassium (K)	17.7^{a}	14.7^{e}	15.3^{de}	16.7^{b}	15.6^{cd}	16.2^{bc}	0.48	<0.001
Sodium (Na)	36.1^{a}	28.1^{c}	30.4^{bc}	33.3^{ab}	31.5^{abc}	31.6^{abc}	1.84	0.031
**Essential trace elements (µg/kg)**								
Copper (Cu)	0.17^{b}	0.18^{b}	0.23^{a}	0.18^{b}	0.14^{c}	0.16^{b}	0.010	<0.001
Iron (Fe)	0.04^{c}	0.05^{b}	0.05^{b}	0.05^{b}	0.01^{d}	0.05^{a}	0.002	<0.001
Iodine (I)	52.20^{b}	54.19^{ab}	55.65^{a}	33.96^{c}	34.91^{c}	36.79^{c}	1.34	<0.001
Manganese (Mn)	0.03^{d}	0.04^{c}	0.04^{b}	0.04^{b}	0.03^{d}	0.05^{a}	0.001	<0.001
Molybdenum (Mo)	5.06^{e}	6.15^{d}	7.92^{b}	8.99^{a}	4.31^{f}	6.83^{c}	0.207	<0.001
Nickel (Ni)	0.02^{b}	0.02^{b}	0.03^{b}	0.10^{a}	0.02^{b}	0.01^{b}	0.013	<0.001
Selenium (Se)	6.50^{b}	6.63^{b}	7.38^{a}	6.88^{b}	5.55^{c}	6.83^{b}	0.203	<0.001
Zinc (Zn)	6.8^{e}	8.0^{cd}	10.3^{a}	8.5^{bc}	7.5^{d}	8.6^{b}	0.21	<0.001
**Non-essential trace elements (µg/kg)**								
Aluminum (Al)	0.02^{c}	0.02^{c}	0.05^{a}	0.03^{b}	0.01^{d}	0.04^{b}	0.002	<0.001
Cobalt (Co)	0.04^{c}	0.03^{c}	0.06^{a}	0.04^{b}	0.03^{d}	0.04^{c}	0.002	<0.001
**Heavy metals (µg/kg)**								
Arsenic (As)	0.13^{c}	0.15^{c}	0.29^{a}	0.25^{b}	0.15^{c}	0.24^{b}	0.017	<0.001

[1] Significances were declared at $P < 0.05$. Means for diet treatment within a row with different letters are significantly different according to Fisher's Least Significant Difference test ($P < 0.05$)

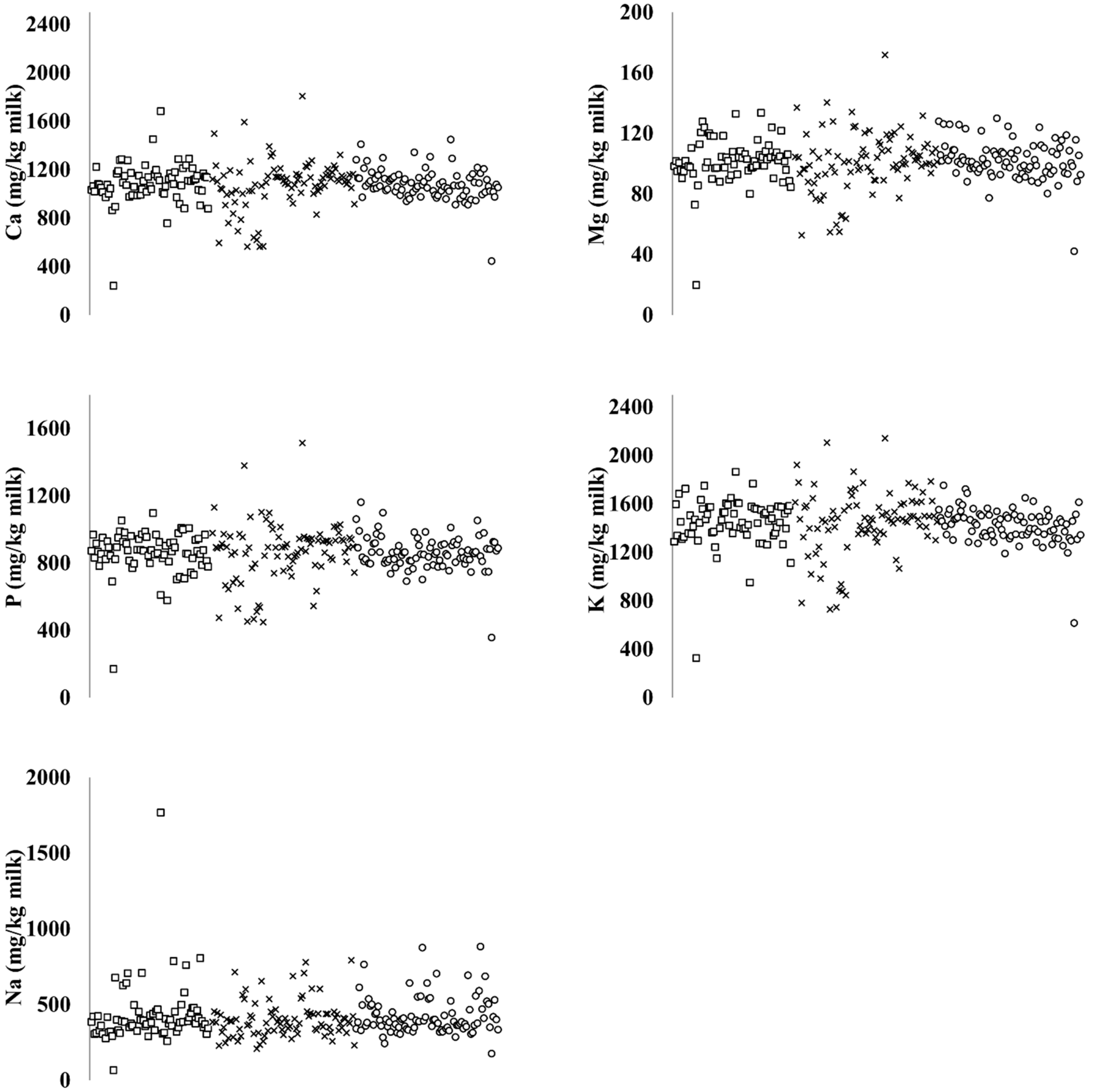

Figure S1

Scatter plots of all measurements of macromineral concentrations in milk samples collected throughout the study from the three experimental groups (▢, control, no seaweed; ×, Low seaweed, 0.75% concentrate DM; ◯, High Seaweed, 1.5% concentrate DM).

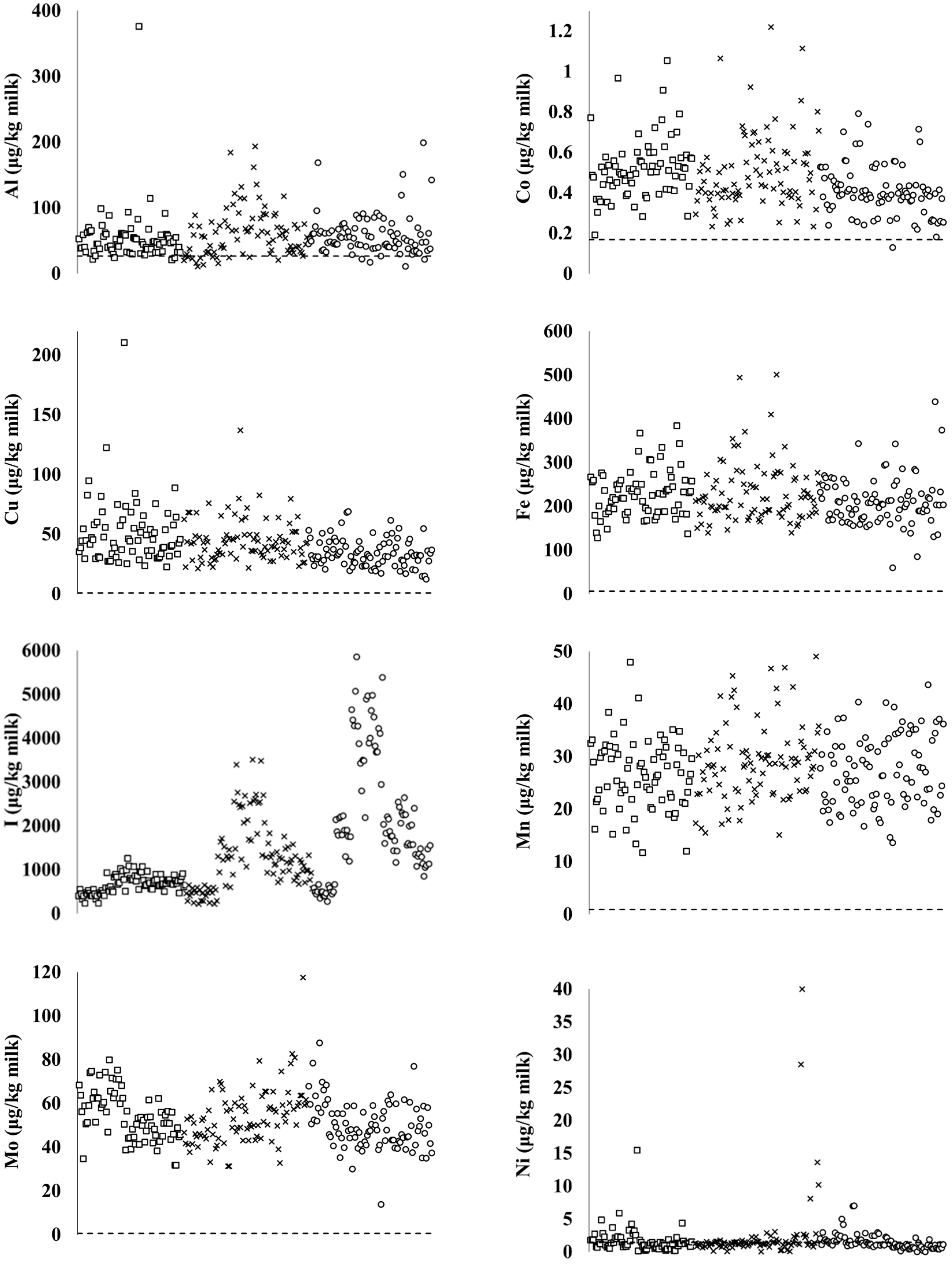
Al (µg/kg milk)
Co (µg/kg milk)
Cu (µg/kg milk)
Fe (µg/kg milk)
I (µg/kg milk)
Mn (µg/kg milk)
Mo (µg/kg milk)
Ni (µg/kg milk)

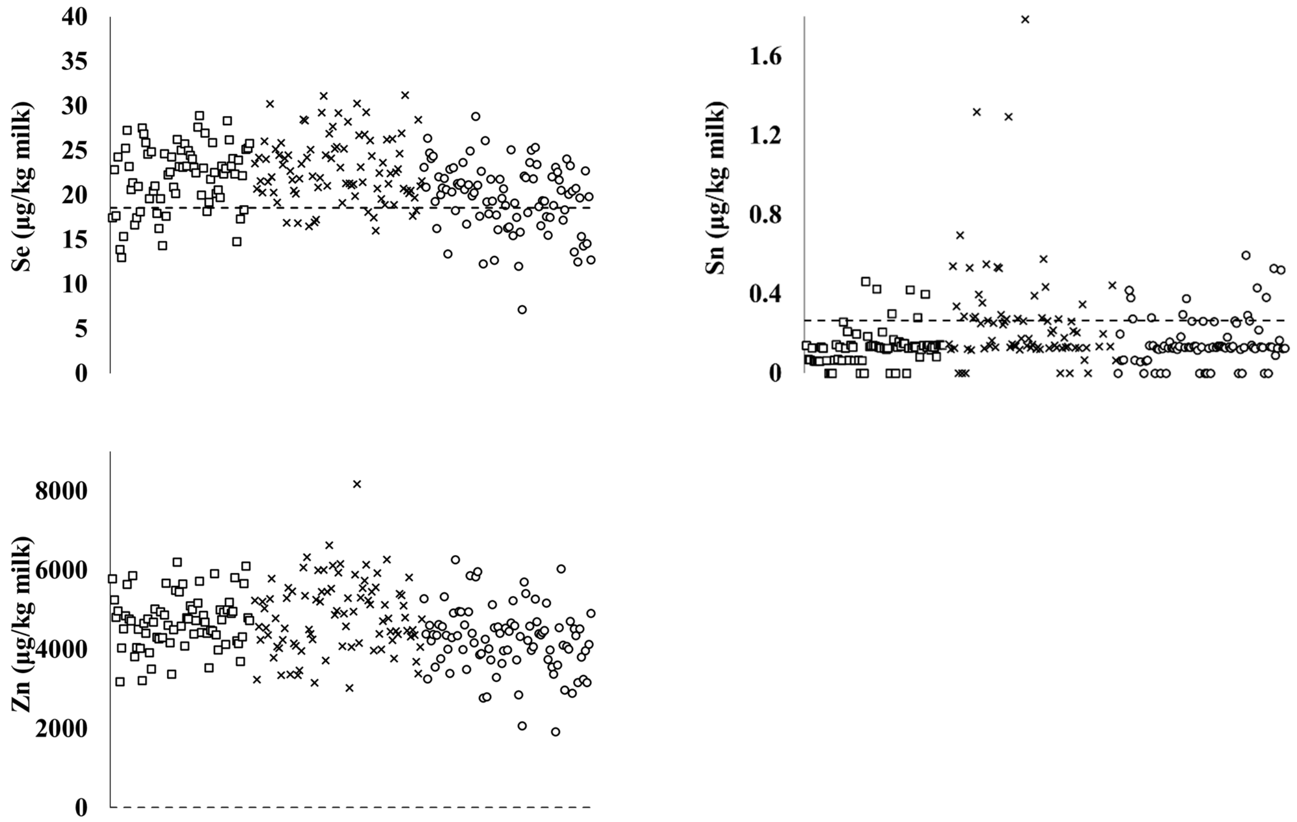

Figure S2

Scatter plots of all measurements of trace element concentrations in milk samples collected throughout the study from the three experimental groups (▢, control, no seaweed; ×, Low seaweed, 0.75% concentrate DM; ◯, High Seaweed, 1.5% concentrate DM). The horizontal dotted lines represent limits of quantification for each element. The limits of quantification of trace elements are: Co, 0.169 µg/kg milk; Cu, 0.911 µg/kg milk; Fe, 6.417 µg/kg milk; Mn, 0.936 µg/kg milk; Mo, 0.625 µg/kg milk; Se, 18.599 µg/kg milk; Zn, 9.974 µg/kg milk; Al, 26.570 µg/kg milk; Ni, 1.457 µg/kg milk; Sn, 0.266 µg/kg milk. The proportions of individual measurements which were below LOQs were: Co, 1%; Cu, 0%; Fe, 0%; Mn, 0%; Mo, 0%; Se, 22%; Zn, 0%; Al, 9%; Ni, 53%; Sn, 88%.

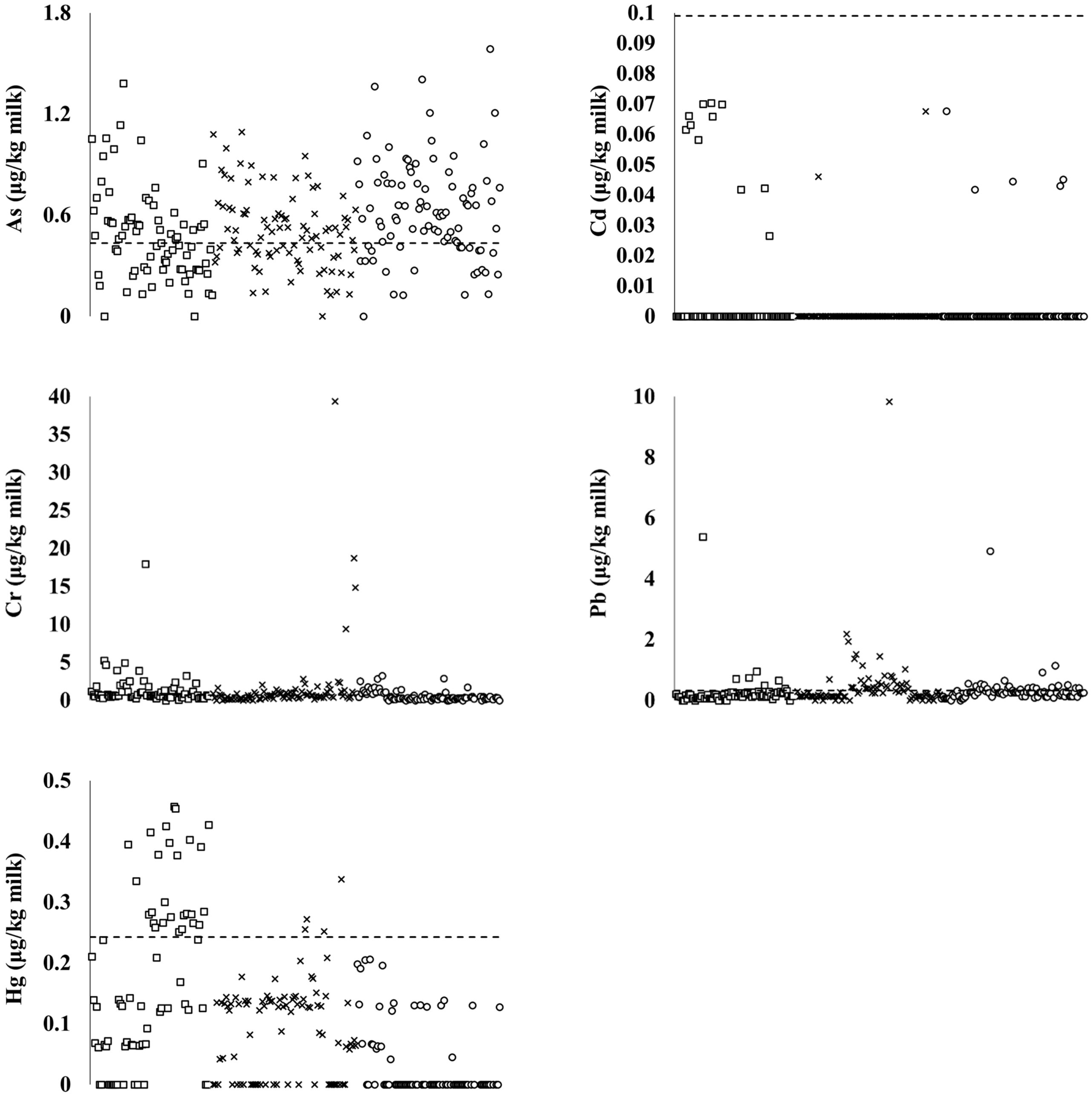

Figure S3

Scatter plots of all measurements of heavy metal concentrations in milk samples collected throughout the study from the three experimental groups (▢, control, no seaweed; ×, Low seaweed, 0.75% concentrate DM; ◯, High Seaweed, 1.5% concentrate DM). The horizontal dotted lines represent limits of quantification for each element. The limits of quantification of heavy metals are: As, 0.435 µg/kg milk; Cd, 0.099 µg/kg milk; Cr, 0.696 µg/kg milk; Hg, 0.243 µg/kg milk; Pb, 0.335 µg/kg milk. The proportions of individual measurements which were below LOQs were: As, 28%; Cd, 96%; Cr, 59%; Hg, 92%; Pb 82%.
